# A targeted proteomic multiplex CSF assay identifies increased malate dehydrogenase and other neurodegenerative biomarkers in individuals with Alzheimer's disease pathology

**DOI:** 10.1038/tp.2016.194

**Published:** 2016-11-15

**Authors:** R W Paterson, W E Heywood, A J Heslegrave, N K Magdalinou, U Andreasson, E Sirka, E Bliss, C F Slattery, J Toombs, J Svensson, P Johansson, N C Fox, H Zetterberg, K Mills, J M Schott

**Affiliations:** 1 Dementia Research Centre, Institute of Neurology, University College London, London, UK; 2 Centre for Translational Omics, Genetics and Genomic Medicine Programme, Institute of Child Health, University College London, London, UK; 3 Department of Molecular Neuroscience, Institute of Neurology, University College London, London, UK; 4 Lila Weston Institute, University College London Institute of Neurology, London, UK; 5 Clinical Neurochemistry Laboratory, Institute of Neuroscience and Physiology, The Sahlgrenska Academy at University of Gothenburg, Sahlgrenska University Hospital, Mölndal, Sweden; 6 Institute of Medicine, Sahlgrenska Academy, University of Gothenburg, Gothenburg, Sweden; 7 Department of Endocrinology, Skaraborg Central Hospital, Skövde, Sweden; 8 Department of Neuropsychiatry, Skaraborg Central Hospital, Falköping, Sweden

## Abstract

Alzheimer's disease (AD) is the most common cause of dementia. Biomarkers are required to identify individuals in the preclinical phase, explain phenotypic diversity, measure progression and estimate prognosis. The development of assays to validate candidate biomarkers is costly and time-consuming. Targeted proteomics is an attractive means of quantifying novel proteins in cerebrospinal and other fluids, and has potential to help overcome this bottleneck in biomarker development. We used a previously validated multiplexed 10-min, targeted proteomic assay to assess 54 candidate cerebrospinal fluid (CSF) biomarkers in two independent cohorts comprising individuals with neurodegenerative dementias and healthy controls. Individuals were classified as ‘AD' or ‘non-AD' on the basis of their CSF T-tau and amyloid Aβ1–42 profile measured using enzyme-linked immunosorbent assay; biomarkers of interest were compared using univariate and multivariate analyses. In all, 35/31 individuals in Cohort 1 and 46/36 in Cohort 2 fulfilled criteria for AD/non-AD profile CSF, respectively. After adjustment for multiple comparisons, five proteins were elevated significantly in AD CSF compared with non-AD CSF in both cohorts: malate dehydrogenase; total APOE; chitinase-3-like protein 1 (YKL-40); osteopontin and cystatin C. In an independent multivariate orthogonal projection to latent structures discriminant analysis (OPLS-DA), these proteins were also identified as major contributors to the separation between AD and non-AD in both cohorts. Independent of CSF Aβ1–42 and tau, a combination of these biomarkers differentiated AD and non-AD with an area under curve (AUC)=0.88. This targeted proteomic multiple reaction monitoring (MRM)-based assay can simultaneously and rapidly measure multiple candidate CSF biomarkers. Applying this technique to AD we demonstrate differences in proteins involved in glucose metabolism and neuroinflammation that collectively have potential clinical diagnostic utility.

## Introduction

Alzheimer's disease (AD) is the most common major neurodegenerative dementia with a prevalence of epidemic proportions expected in the coming decades.^1^ Biomarkers are increasingly utilised for clinical diagnosis^2^ and are essential for diagnosis in the preclinical phase, which may begin 20 years or more before symptom onset.^3^ Molecular biomarkers currently used in clinical research diagnostic criteria for AD include amyloid positron emission tomography imaging, and cerebrospinal fluid (CSF) β-amyloid 1–42 and tau, which reflect the key hallmarks of AD pathology, that is, amyloid plaques and neurofibrillary tangles.^3, 4^ Although these biomarkers can distinguish AD pathology from non-AD pathology with reasonable sensitivity and specificity,^5^ there remains a need for new biomarkers,^6^ including those that can detect pathological changes before overt neuronal death; correlate with the progression of neurodegeneration for clinical trials; explain phenotypic diversity;^7^ and allow for accurate prognostication.

Over recent years, a large number of candidate biomarkers have been identified, particularly in CSF, reflecting a range of pathophysiological processes including cholesterol metabolism, neuroinflammation and amyloid processing.^6^ However, to date few, if any, have been adopted in clinical practise. This is, in part, because of the time taken to develop suitable immunoassays; availability of biomarker multiplex panels; replicability of immunoassays, with very few novel biomarkers being successfully validated in large independent cohorts.^8^ Mass spectrometry can measure a large number of potential biomarkers (reviewed by Kroksveen *et al.*^9^ and Brinkmalm *et al.*^10^) and therefore has considerable potential utility for the identification of new biomarkers, and for use in clinical practice. However, most mass spectrometry studies in AD have largely focused on biomarkers for which there is already an immunoassay;^11^ and, although mass spectrometry has considerable potential clinical utility, this has been limited in part due to the lack of a streamlined, cost-effective pipeline to rapidly test large numbers of potential biomarkers concurrently.

Recently, our group and others have developed targeted proteomics methods using liquid chromatography–tandem mass spectrometry to multiplex scores of peptides in a single rapid CSF assay, which has low technical variability^11^ and relatively low cost. We have applied this to clinical cohorts of patients with Parkinson's disease and Dementia with Lewy Bodies,^12^^,^^13^ and a previous study has used similar technology to assess CSF biomarkers of progression in a small number of AD subjects longitudinally.^14^ Such assays allow quantification of proteins^15^ with high reproducibility^16^ and thus have potential utility in facilitating the rapid validation of biomarkers in clinical cohorts overcoming a bottleneck in biomarker development.

The aims of this study were to (a) evaluate the feasibility of this rapid ‘one pot', multiplexed, targeted proteomic assay to measure biomarkers of interest in clinical cohorts of individuals with AD, other degenerative diseases and healthy controls and (b) explore differences in novel biomarker concentrations between individuals with AD and non-AD classified according to their CSF tau and β-amyloid levels.

## Materials and methods

### Ethics statement

The study was conducted in accordance with local clinical research regulations and was approved by the local Queen Square Ethics Committee. Where appropriate, individuals gave informed written consent.

### Subjects and CSF collection

#### Cohort 1

This cohort included 107 individuals, 88 undergoing investigation for cognitive concerns and 19 healthy age-matched controls without cognitive concerns. The majority of subjects were from a single memory centre at Skaraborg hospital in Sweden (*n*=78) and this cohort has previously been described in detail.^17^ A further 29 CSF samples from individuals with cognitive concerns from another single memory centre in Sweden were included. Healthy control participants had an lumbar puncture for research purposes only; they were asymptomatic spouses of affected individuals or healthy controls without subjective cognitive concerns.

#### Cohort 2

This cohort included 92 individuals assessed at the Specialist Cognitive Disorders Service at Queen Square, London, UK between 2011 and 2014. All subjects had a clinical CSF examination as part of their diagnostic work-up. Twenty-six asymptomatic controls (spouses of research participants) were also included; these individuals had no cognitive concerns and had lumbar punctures for research purposes only. For the patient group, we recorded the nearest Mini-Mental State Examination (MMSE) score to the date of the lumbar puncture. Rate of cognitive decline was estimated using the formula (30-MMSE at time of lumbar puncture/duration of cognitive symptoms in months). *APOE* genotype was determined by measuring peptides corresponding to apoE2, apoE3 and apoE4 in CSF using the multiple reaction monitoring (MRM)-based liquid chromatography–tandem mass spectrometry assay as previously described,^18^ and individuals were classified as *APOE* ɛ4-positive or -negative.

### CSF collection and routine biomarker analysis

For all subjects, CSF was collected by lumbar puncture in polypropylene containers, and was spun at 300 *g* for 10 min at 4 °C and the supernatant was frozen in aliquots at −80 °C within 60 min. CSF levels of β-amyloid (1–42), T-tau and P-tau were analysed using INNOTEST enzyme-linked immunosorbent assays (ELISAs) (Fujirebio Europe, Gent, Belgium) according to the manufacturer's protocols.

### Neurochemical classification

We classified each individual independent of clinical diagnosis on the basis of CSF profile. A previous study has shown that a tau/β-amyloid (1–42) ratio cutoff of 0.52 gives a sensitivity of ~93% and specificity of ~83% for AD diagnosed clinically;^19^ moreover, according to the manufacturer's guidelines, a P-tau of >63 gives a sensitivity of 74% and specificity of 85% for AD compared with other neurodegenerative diseases.^20^ To ensure that the neurochemical AD subjects had AD, we used stringent CSF criteria defined as: tau/β-amyloid (1–42) ratio >1 and P-tau >63; a negative Alzheimer's signature CSF profile was defined by Tau/β-amyloid (1–42) ratio <0.52 and P-Tau<63. As the purpose of this study was to determine biomarkers that differentiate between established AD and healthy controls, we excluded individuals with ‘grey zone' CSF profiles (that is, those with Tau/β-amyloid (1–42) ratio >0.52 and <1.0 or non-compatible P-tau) using discovery and replication cohorts.

### Mass spectrometry

#### Targeted proteomics: MRM-based triple quadrupole mass spectral assay

A multiplexed, 10 min, targeted proteomics assay performed on Waters ultraperformance liquid chromatography system (Manchester, UK) coupled to Waters Xevo TQ-S triple quadrupole mass spectrometer, operated in the MRM mode, was used to detect a panel of 54 biomarkers as described previously.^13^ The panel consisted of proteins that were identified from a literature review (see Supplementary Table 1) and new markers identified from proteomic profiling described previously including four novel markers previously found to be elevated in AD and Dementia with Lewy Bodies compared with controls: malate dehydrogenase (MDH); serum amyloid A4; GM_2-_activator protein and prosaposin.^13^ A standard curve 0–40 pmols per 100 μl CSF of each peptide was analysed in duplicate at the end of the run for quantitation and performance standardisation (see Supplementary Table 1). Twenty nanograms of yeast enolase protein standard (Sigma, Dorset, UK) and 10–50 pmols heavy labelled peptide standards (Thermo Scientific, Loughborough, UK) were added to 100 μl of CSF. CSF was freeze-dried and trypsin-digested as described previously.^21^ A single 35 μl injection of each CSF digest was injected on a Waters CORTECS UPLC C18 + Column, 90 Å, 1.6 μm, 3 mm × 100 mm column attached to a C18+ VanGuard pre-column. Ultra performance liquid chromatography (UPLC) and mass spectrometry tune conditions were performed as described previously.^22^ Dynamic MRM was performed over a 10-min gradient. Quality control (QC) runs of pooled CSF digests were run in triplicate at the start of the run and then every 10 injections. A coefficient of variation (CV) within ±10% for each QC was considered acceptable. CSF was spiked with peptides to create standards with average concentrations of biomarker levels and analysed for intra- and interbatch variation. Chromatograms were analysed using the Waters Targetlynx software. Peptides were standardised by either using a spiked heavy labelled peptide or to a yeast enolase peptide. Absolute levels were obtained from standard curves. Standard curve linearity of *r*^2^ >0.9 was achieved for all calibration curves.^23^ Data were exported to Microsoft Excel (Microsoft, Redmond, WA, USA) and GraphPad Prism (GraphPad Software, La Jolla, CA, USA) for statistical analysis. Intrabatch variation was determined as being between 3.0 and 5.1% and inter-batch variation being 7.6–8.5% (*n*=10, three consecutive days). Investigators were fully blinded to clinical and neurochemical diagnosis during this analysis.

#### Experimental design

**Figure 1:**
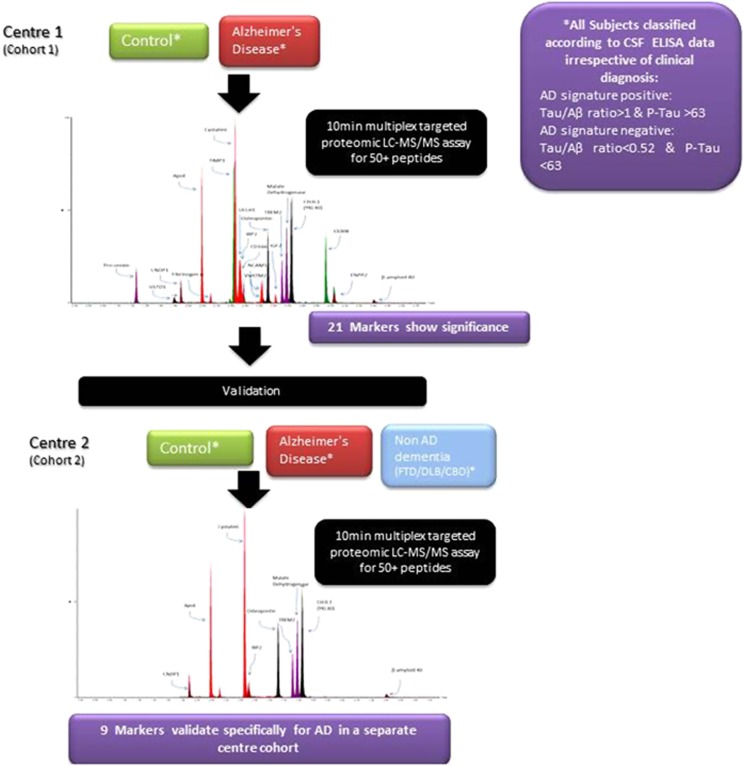
Study design outline (univariate analysis). AD, Alzheimer's disease; CSF, cerebrospinal fluid; ELISA, enzyme-linked immunosorbent assay; LC-MS, liquid chromatography-mass spectrometry.

The experimental design of this study is summarised in Figure 1. The panel of 54 novel markers was first assessed in cohort 1. Markers showing significant differences between the AD-positive/negative groups from this initial analysis were then further assessed in cohort 2.

### Statistical analysis

#### Univariate analysis of proteins of interest

We performed a univariate analysis of all proteins of interest. We determined which individuals in Cohort 1 were Alzheimer-positive and which were Alzheimer-negative based on their CSF neurochemical profile, and compared levels of proteins determined by targeted mass spectrometry using *t*-tests between the positive/negative groups when there were no clear departures from a normal distribution, and Wilcoxon rank-sum tests for skewed or truncated data. Proteins showing statistically significant differences between AD-positive/negative groups in Cohort 1 were then tested in Cohort 2 as a validation set. All analyses were carried out at a significance level of *P*<0.05; to control for the risk of Type 1 error for multiple biomarker comparisons, results were also controlled using the false discovery rate (FDR). 'Validated biomarkers' were those found to separate neurochemically defined AD/non-AD in both data sets at an FDR-corrected significance level of *P*<0.05.

#### Multivariate analysis

Independent of the biomarkers discovered in step 1, we carried out an analysis of the entire targeted mass spectrometry data set to determine which markers contributed the most to the separation between AD and non-AD in each cohort separately. To do this, we used an orthogonal projection to latent structures discriminant analysis (OPLS-DA) implicated in the software SIMCA, Umetrics, Sweden, as previously described,^24^ classifying subjects on the basis of their AD signature (positive/negative) CSF. In brief, this is an algorithm that determines the vector that maximally separates these groups in the multivariate orthogonal space. Non-normally distributed data were log-transformed before analysis.

We used receiver operating characteristic curves to determine the diagnostic utility of the ‘validated' biomarkers from step 1 using the ‘roctab' command in Stata Version 12.1 (Stata, College Station, TX, USA) using the healthy control subjects with a non-AD neurochemical profile from Cohort 2 as the control group. We finally explored the relationship between each of the validated biomarkers and the established CSF biomarkers Tau, P-Tau, and rate of cognitive decline by fitting separate regression models for each of the ‘Validated' biomarkers including all subjects with AD or non-AD CSF in the model, except when exploring the relationship with cognitive function when only individuals with AD CSF were included. Linear regression was used to explore the relationship between novel biomarkers and T-tau, P-Tau, β-amyloid (1–42), MMSE and rate of cognitive decline. Unless otherwise stated, all analyses were carried out using Stata V12.1. Graphs were created using GraphPad prism V5 (Graphpad Software). The correlation matrix was created using Microsoft Excel.

## Results

### Comparing neurochemical AD and non-AD subjects

In Cohort 1, 35 individuals fulfilled CSF neurochemical criteria for AD, and 31 had a non-AD CSF profile. The remaining 41 had an intermediate profile (that is, Tau/β-amyloid (1–42) ratio >0.52 and <1.0 or non-compatible P-Tau) and were not included in further analyses. As expected, there were significantly more *APOE* ɛ4 carriers in the AD group (Table 1A). Groups were well matched for sex; the neurochemical AD group was significantly (~4 years) older than the non-AD group.

In Cohort 2, 46 individuals fulfilled neurochemical CSF criteria for AD, 44/46 of whom had a clinical diagnosis of AD and thus fulfilled contemporary (International Working Group (IWG-2)^2^ and National Institute of Aging (NIA)^4^) criteria for AD; the remaining two were controls. Of the 36 subjects with non-AD CSF, 22 were healthy controls, seven had subjective cognitive concerns and the others were diagnosed with other non-AD neurodegenerative dementias including semantic dementia, behavioural variant frontotemporal dementia and Lewy Body dementia. A further 10 individuals had an intermediate profile and were not included in further analyses. Groups were well matched for age and sex. As expected, there were significant differences in MMSE and *APOE* status. CSF ELISA biomarker data are given in Table 1B.

### Univariate analysis: comparing neurochemical AD and non-AD subjects

**Figure 2:**
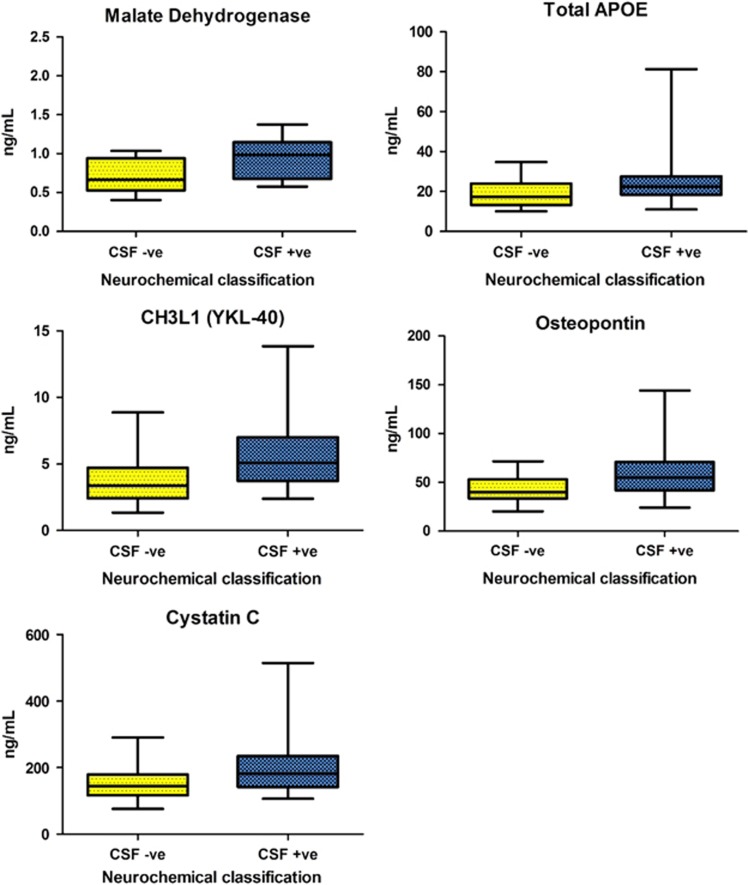
Boxplots and whiskers (representing 10th and 90th percentiles) comparing Alzheimer's disease (AD) and non-AD cerebrospinal fluid (CSF) concentrations of proteins surviving false discovery rate (FDR) correction in the univariate analysis of cohort 2.

Comparing the neurochemically defined AD and non-AD groups in Cohort 1, there were significant differences in measured biomarker concentrations in 21 markers, of which 15 survived FDR correction (Table 2A). Taking these 15 proteins forward to the validation cohort (Cohort 2), 9 markers (total apoE (which refers to the APOE protein where the peptide is taken from a conserved region of ApoE and quantitate irrespective of isoform status), β-amyloid40, Carnosine Dipeptidase 1, cystatin C, insulin-like growth factor-binding protein 2, MDH, osteopontin, triggering receptor expressed on myeloid cells 2 and YKL-40) were significantly elevated in the patients with both clinically and neurochemically defined AD. Five biomarkers (total apoE, cystatin C, MDH, osteopontin and YKL-40) survived FDR correction in both the test (Cohort 1) and validation (Cohort 2) sets and were defined as 'validated biomarkers' (Figure 2).

We also compared the AD (CSF +ve) and non-AD (CSF –ve) dementias in Cohort 2 excluding healthy control subjects. A similar list of 16 markers was significantly different between the groups, with only MDH surviving FDR correction (Table 2B).

### Multivariate analysis classified according to clinical diagnosis and neurochemical diagnosis

**Figure 3:**
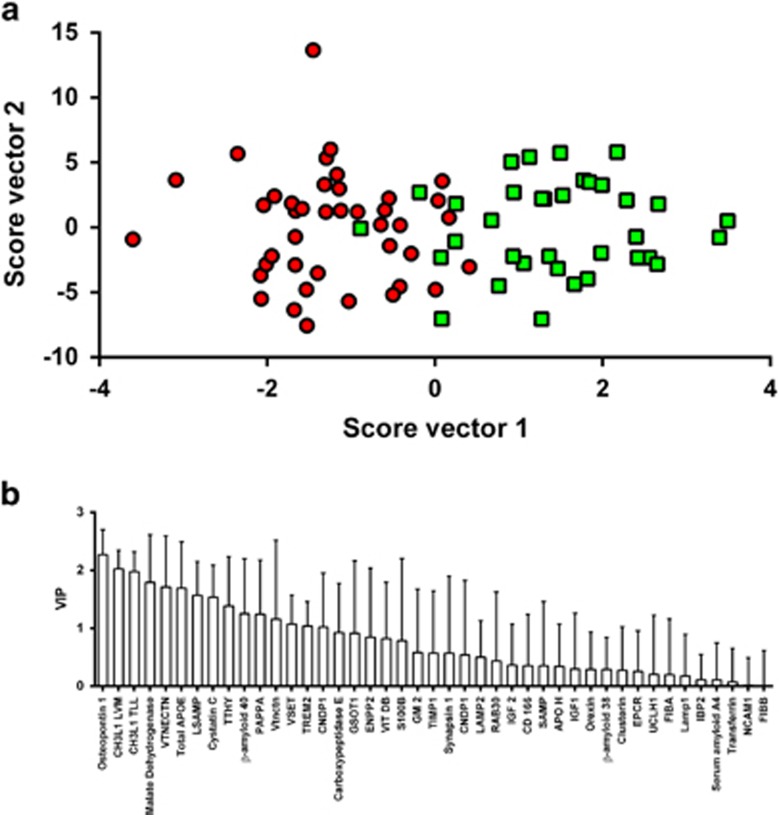
(**a**) Orthogonal projection to latent structures discriminant analysis (OPLS-DA) analysis using data from cohort 2. Subjects are colour-coded according to neurochemical status: red circles=Alzheimer's disease (AD); green squares=non-AD. The corresponding *R*^2^ and *Q*^2^ values for the model were 0.56 and 0.3, respectively. (**b**) Variable importance on projection plot corresponding to the score plot in **a**.

Results of the OPLS-DA analysis using Cohort 2 are shown in Figure 3. Peptides corresponding to the following biomarkers were identified as the seven strongest predictors of group membership when separating the groups on neurochemical diagnosis (AD profile-positive; AD profile-negative): osteopontin, YKL-40, MDH, vitronectin, total apoE, limbic system-associated membrane protein and cystatin C. Osteopontin and YKL-40 also topped the list for cohort 1 (data not shown).

### Diagnostic utility

When applied to Cohort 2 for which full clinical data were available, the five 'validated biomarkers' could individually differentiate AD from non-AD healthy control CSF with areas under the curve (AUC) as follows: total apoE=0.62; cystatin C=0.62; MDH=0.67; osteopontin=0.79; and YKL-40=0.75. In a multivariate logistic regression analysis including all of these variables, the combination could differentiate AD from non-AD healthy control CSF with an AUC of 0.88. When we included all individuals in cohort 2, including those with grey-zone CSF profiles and classified them by clinical diagnosis only, the combination of biomarkers could differentiate AD from non-AD neurodegeneration with an AUC=0.7.

### Correlation of proteins with each other and existing CSF biomarkers

**Figure 4:**
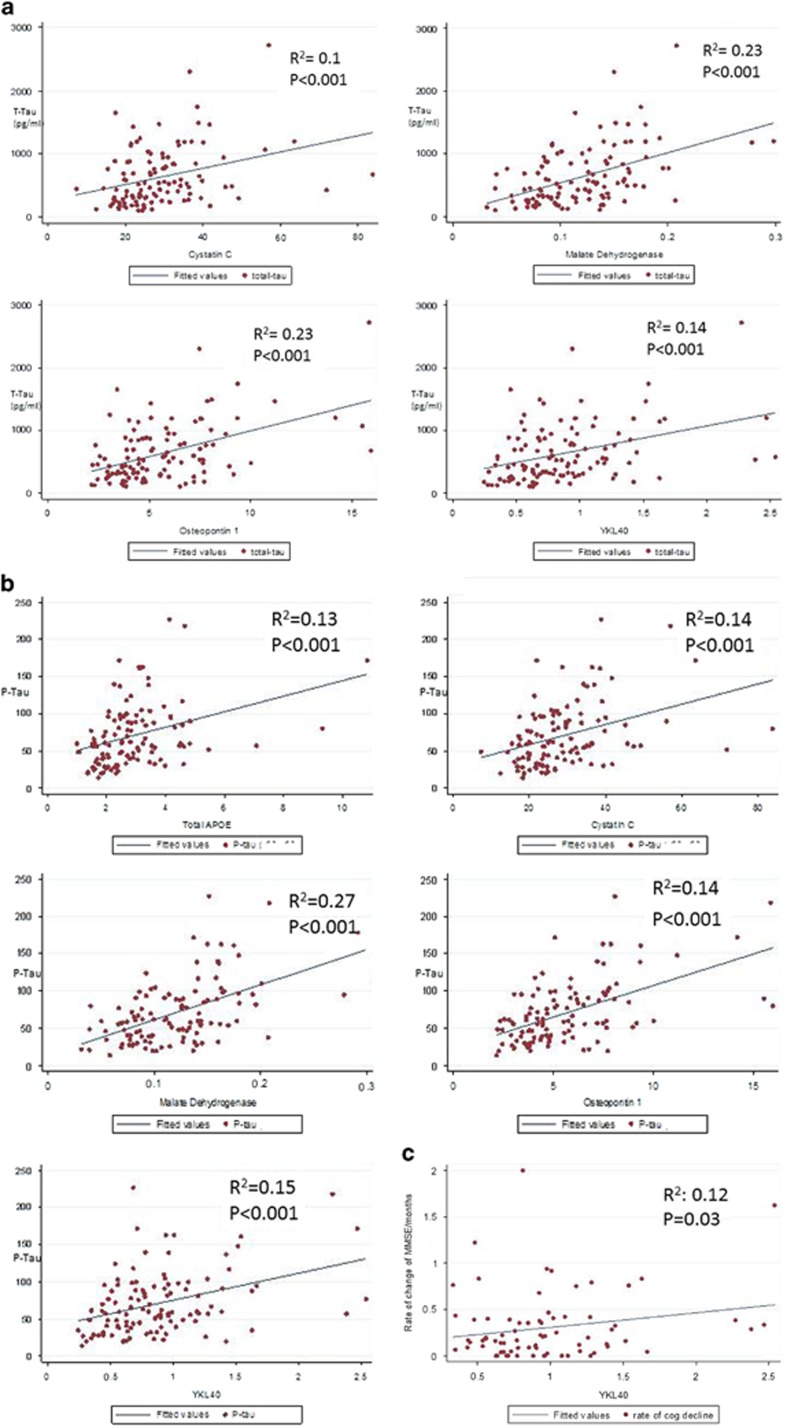
(**a**) Scatter plots showing correlations between cerebrospinal fluid (CSF) T-Tau (enzyme-linked immunosorbent assay, ELISA) and 'validated biomarkers' measured using targeted proteomics using subjects in Cohort 2. (**b**) Scatterplots showing correlations between CSF P-Tau (ELISA, pg ml^−1^) and 'validated biomarkers' measured using targeted proteomics using subjects in Cohort 2. (**c**) Scatterplots showing correlations between rate of cognitive decline (30-Mini-Mental State Examination (MMSE) score/duration of cognitive symptoms in months) and 'validated biomarkers' measured using targeted proteomics using subjects in Cohort 2.

**Figure 5:**
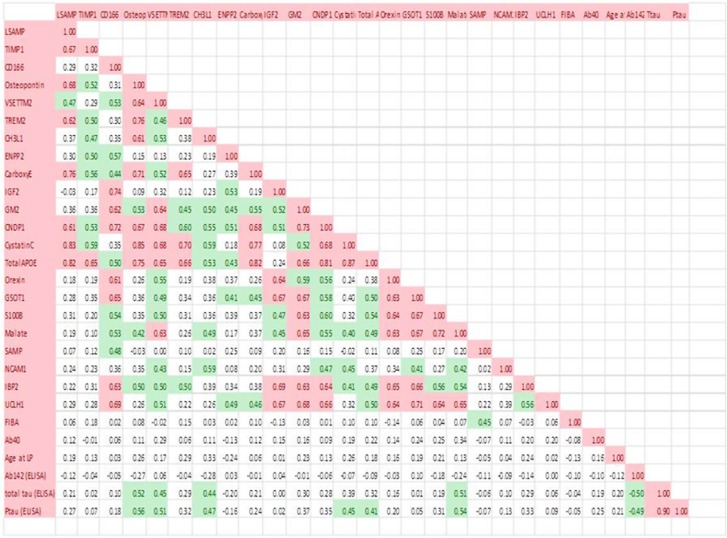
Correlation matrix including all biomarkers listed in Tables 2A and 2B and enzyme-linked immunosorbent assay (ELISA) data for β-amyloid 1-42

To explore the relationship between established CSF biomarkers measured using ELISA and the proteins measured using this targeted proteomics assay, regression analyses were carried out between each of the five validated biomarkers and β-amyloid 1–42, T-Tau and P-Tau including all subjects in the analysis irrespective of the neurochemical status. None were significantly correlated with age or β-amyloid 1–42. Cystatin C, MDH, osteopontin and YKL-40 were each correlated with both T-Tau and P-Tau (Figures 4a and b). A correlation map shows which of the proteins from Tables 2A and 2B were correlated with one another (Figure 5).

In a regression analysis including age, sex and APOE status in the model, there was a weak association between YKL-40 and rate of cognitive decline in the AD cohort (Figure 4c). There were no other significant associations between proteins measured using this targeted proteomics assay and rate of cognitive decline.

## Discussion

In this study we use a targeted, fully quantitative multiplexed assay to measure a panel of 54 proteins identified in previous studies as of potential interest in AD and neurodegeneration. We show that this ‘one-pot' test, which requires a very small volume of CSF (100 μl), can be used to rapidly validate biomarkers of potential interest in clinical cohorts.

We identified five biomarkers that differentiate neurochemical AD from non-AD in two independent clinical populations from different centres, all of which were also identified as those markers contributing most to the separation in an independent multivariate model differentiating by neurochemical AD/non-AD. These include markers of neuroinflammation, that is YKL-40, cystatin C and osteopontin; total apoE, the best recognised genetic risk factors for AD; and MDH, a key enzyme in brain glucose metabolism. We compared AD CSF with other suspected non-AD neurodegenerative subjects and, although with the caveat that sample sizes are small, MDH was also significantly higher in the AD cohort, suggesting that it could be specific to AD neurodegeneration. Whereas the majority of these biomarkers are unlikely to have diagnostic utility individually as they have lower sensitivity/specificity than T-Tau/β-amyloid 1–42 ratio or P-Tau, MDH, YKL-40 and osteopontin were individually capable of differentiating AD from non-AD CSF with AUC⩾0.75; and collectively all five of the 'validated' biomarkers could distinguish individuals with AD-positive/negative CSF with AUC=0.88.

The biomarkers identified all have potentially important roles in AD pathogenesis. MDH is one of eight mitochondrial enzymes involved in the tricarboxylic acid cycle, the main pathway for oxidation of glucose in the brain. Deficits in brain glucose metabolism and oxidative stress are now recognised in AD pathophysiology,^25^ and MDH is found in increased concentrations in the cortex and hippocampi of AD brains of humans and mice at autopsy compared with healthy controls,^26, 27, 28^ whereas other enzymes in the cycle are reduced or unchanged.^27^ The mechanism for increased CSF MDH is unclear; however, from studies of other pathological brain conditions (ischaemia, hypoglycaemia and thiamine deficiency), anabolic catabolism of glucose may occur as an alternative mitochondrial energy-generating pathway^29^ and induce cell death.^25^ To our knowledge this is the first *in vivo* evidence that glucose metabolism in altered in AD CSF. In this context, it is notable that glucose hypometabolism measured using fludeoxyglucose positron emission tomography predates cognitive symptoms and is correlated with cognitive function in AD.^30^

Cystatin C colocalises with amyloid and is involved in microglial activation.^31^ Several previous biomarker discovery studies have compared concentrations of cystatin C in AD and control CSF using ELISA, sometimes with equivocal or conflicting results.^32, 33, 34^ Cystatin C has also been identified using mass spectrometry in biomarker discovery studies of AD CSF.^35, 36^ Our findings replicate these results in two further independent cohorts, suggesting that mass spectrometry may be a more sensitive and reproducible method for quantifying this protein. Furthermore, CSF cystatin C predicts rate of brain atrophy, a surrogate marker of neurodegeneration, in established and prodromal AD.^37^

Osteopontin is a cytokine expressed by cytotoxic T cells and is involved in macrophage recruitment and activation. It is increased in pyramidal neurons in AD,^38^ AD transgenic mouse models,^39^ elevated in human AD CSF^40, 41^ as well as CSF of familial AD mutation carrying individuals.^42^ Khan *et al.*^43^ identified osteopontin as one of the top three proteins differentiating AD and control CSF using a multivariate support vector machine algorithm on data from Alzheimer's Disease Neuroimaging Initiative. Although differences in osteopontin were not found between AD and controls in another mass spectrometry assay, it was identified as a predictor of conversion from mild cognitive impairment to AD.^44^ Using mass spectrometry assays we have now found osteopontin to be elevated in AD in two independent cohorts and individuals with Lewy Body dementia, many of whom will have AD pathology, we suggest this is likely to be a real finding.

YKL-40 is expressed by microglia and astrocytes in the brain and is implicated in the neuroinflammatory response to β-amyloid deposition.^45^ Elevated CSF YKL-40 is seen in a number of neurodegenerative diseases including prodromal AD,^45^ as well as in stroke and multiple sclerosis. It was identified previously as a potential AD biomarker in an unbiased liquid chromatography-mass spectrometry biomarker discovery study comparing CSF from individuals with AD to controls^46^ and was higher in AD CSF in another targeted proteomics study.^14^ Although there are commercially available immunoassays for YKL-40 and it is unlikely to be specific for AD, it could prove a useful marker in the context of a multiplexed panel of CSF markers of neuroinflammation, which might improve diagnostic accuracy or help predict rate of disease progression. It has previously been shown that concentrations are correlated with AD disease progression,^47^ which these findings support, suggesting that it could also be a meaningful functional biomarker.

As described previously, assays of this type can measure peptides corresponding to apoE isoforms E3, E4 and E2 accurately enough to determine APOE genotype,^48^ which could have significant practical and financial benefits. However, the utility of CSF total apoE concentration is less well established, with previous non-mass spectrometry studies showing no clear difference in concentration between AD and control CSF.^48, 49^ Our finding that total apoE levels differentiated between all non-AD cases (including controls) and non-AD neurodegenerative cases (excluding controls) suggests that it may be a biomarker with specificity for AD.

This study has a number of strengths, notably the use of two independent cohorts allowing for discovery/replication, conservative statistical approaches correcting for multiple comparison and two independent techniques for assessing biomarker differences between groups. Subjects were recruited prospectively and samples were collected according to a standard operating protocol^50^ to minimise the influence of pre-analytical factors on biomarker profile. Although detailed clinical data were available for some but not all of the test cohort (Cohort 1), as described previously,^17^ the validation cohort (Cohort 2) was well characterised and matched for age and sex, and were from a single centre. Individuals in the AD group were relatively young, reflecting our clinical focus and that younger individuals are more likely to be referred for diagnostic lumbar puncture.^51^ As the design of this study was to determine whether the assay could differentiate between AD and non-AD pathology, groups were defined by CSF neurochemical status and we chose not to classify by clinical diagnosis, except when determining clinical utility. Even in specialist centres clinical diagnostic accuracy can be variable;^52, 53, 54, 55^ a combination of CSF tau and β-amyloid can predict pathological diagnosis with a sensitivity and specificity of ~90%^5^ in individuals whose brains were subsequently examined postmortem. The neurochemical non-AD group was mixed; 61% were controls, whereas the other 39% were concerned about their cognition and may have had another neurodegenerative disease. This study is therefore likely to identify biomarker associated with AD and may not be capable of detecting other markers of neurodegeneration, which may also be altered in the non-AD CSF group. Finally, as well as being highly selective and specific,^56^ and with a wide dynamic range^57, 58^ MRM is still likely to be as sensitive as ELISA, which is currently considered the gold standard for protein detection.^59^

To date, a large number of candidate CSF proteins have been suggested as potential biomarkers for presymptomatic AD based on biomarker discovery experiments in asymptomatic individuals carrying an autosomal-dominant mutation for AD.^42^ Blood-based biomarkers have also been identified from twin studies^60^ where some individuals subsequently develop cognitive impairment. This type of MRM assay has potential to investigate candidate biomarkers of preclinical disease in months rather than the years that it might take to develop an ELISA-based assay with the added benefit that the reagent costs, which might be substantial for a novel immunoassay, are negligible.^61^

A previous study of AD, mild cognitive impairment and control CSF^62^ used a similar pipeline to validate a panel of biomarkers in a single cohort with longitudinal CSF samples, and found four biomarkers that differentiated clinical AD from healthy controls, including YKL-40, Complement component C3, transthyretin and amyloid A4 protein. YKL-40 was identified in our OPLS-DA analysis and univariate analysis comparing neurochemical AD to non-AD. Similarly, transthyretin was identified in AD and mild cognitive impairment CSF^62^ and in our OPLS-DA analysis; serum amyloid A4 protein contributed to variance in our OPLS-DA analysis; complement component C3, however, was not included on our panel. Our study uses a larger panel of biomarkers and has some methodological advantages: the assay is significantly shorter and simpler; samples do not require to be aliquoted into multiple small volumes and can be analysed from one ‘single pot', and therefore lends itself extremely well to multiplexing large numbers of peptides.

## Conclusions

We describe a streamlined and efficient mass spectrometry technique for measuring multiple CSF biomarkers concurrently, and using this methodology validate a number of biomarkers including markers of neuroinflammation and glucose metabolism that distinguish AD CSF from controls. This highly specific method offers the opportunity to validate large numbers of candidate biomarkers in very small volumes of CSF with negligible reagent costs, and is ideally suited both for biomarker discovery, and for translation into a rapid and cost-effective clinical test.

## Figures and Tables

**Table 1A:** Demographics and CSF profiles of individuals from Cohort 1

	*Neurochemical AD (*N*=35)*	*Neurochemical Non-AD (*N*=31)*	*AD* versus *non-AD (*P*-value)*
Sex (% male)	42.9	64.5	0.09
% APOE4 heterozygotes	42.9	22.6	<0.001
% APOE4 homozygotes	25.7	3.2	<0.001
% APOE2 heterozygotes	5.7	19.4	<0.001
Aβ1-42 (pg ml^−1^)	453±147	907±221	<0.001
T-tau (pg ml^−1^)^a^	654 (505–969)	255 (210–294)	<0.001
P-Tau (pg l^−1^)	119.7±72.4	44.5±12.0	<0.001
Tau/Aβ1-42 ratio^a^	1.51 (1.25–2.06)	0.25 (0.22–0.34)	<0.001

Abbreviations: AD, Alzheimer's disease; CSF, cerebrospinal fluid.

Data are shown as mean±s.d., unless otherwise stated

.

Log-transformed for regression analyses; values quoted as the median (interquartile range).

**Table 1B:** Demographics and CSF profiles of individuals from Cohort 2

	*Neurochemical AD (*n*=46)*	*Neurochemical Non-AD* *(*n*=36)*	*AD* vs *Non-AD (*P*-value)*
Age at lumbar puncture	62.9±8.0	58.5±8.8	0.2
Sex (% male)	39.1	44.4	0.5
MMSE	20.6±5.6	26.7±6.9	<0.001
Duration of cognitive symptoms (months)	36.4±17.4	NA	NA
Rate of cognitive decline (MMSE points per month)	0.36±0.42	NA	NA
% Individuals fulfilling McKhann criteria	95.7	0	<0.001
% APOE ɛ4-positive	67.4	33.4	<0.001
Aβ1-42 (pg ml^−1^)	408±168	960±291	<0.001
T-tau (pg ml^−1^)^a^	947 (760–1196)	234.5 (174.5–315.5)	<0.001
P-Tau (pg ml^−1^)	107.5±38.12	35.5±13.2	<0.001
Tau/Aβ1-42 ratio^a^	2.5 (1.8–4.1)	0.25 (0.19–0.33)	<0.001

Abbreviations: AD, Alzheimer's disease; CSF, cerebrospinal fluid; MMSE, Mini-Mental State Examination; NA, not applicable.

Data are shown as mean±s.d., unless otherwise stated.

Log-transformed for regression analyses and values quoted as the median (interquartile range).

**Table 2A:** Univariate analysis comparing biomarkers in AD and non-AD CSF from Cohort 2

	P*-value (cohort 1)*	P-*value (cohort 2)*	*Fold change in cohort 2*
**Malate dehydrogenase**^a^	0.005*	<0.001*	**2.12**
**Total APOE**^a^	<0.001*	0.005*	**1.55**
**Chitinase-3-like protein 1(YKL-40)**^a^	<0.001*	<0.001*	**1.52**
**Osteopontin**^a^	<0.001*	<0.001*	**1.50**
NCAM1	0.03	0.38	1.40
UCLH1	0.003*	0.88	1.30
**Cystatin C**^a^	0.008*	0.003*	**1.28**
*Beta-amyloid 40*	<0.001*	*0.01*	*1.28*
*CNDP1*	0.01*	*0.03*	*1.26*
V-Set and transmembrane domain containing protein 2A	0.03	0.06	1.25
Fibrinogen A	0.03*	0.83	1.24
*IBP-2*	0.007*	*0.04*	*1.20*
S100B	<0.001*	0.06	1.20
TREM2	0.001*	0.05	1.18
Serum amyloid p-component	0.007*	0.33	1.14
CD166	0.03	0.25	1.12
Pro-orexin	<0.001	0.22	1.11
TIMP metallopeptidase inhibitor 1	0.03	0.5	1.05
IGF2	0.005*	0.72	0.97
Glutathione-S-transferase omega-1	0.006*	0.75	0.91
ENPP2	0.05	0.11	0.89

Abbreviations: AD, Alzheimer's disease; CNDP1, carnosine dipeptidase 1; CSF, cerebrospinal fluid; FDR, false discovery rate; IBP-2, insulin-like growth factor-binding protein 2; IGF2, insulin-like growth factor 2; NCAM1, neural cell adhesion molecule 1; OPLS-DA, orthogonal projection to latent structures discriminant analysis; TREM2, triggering receptor expressed on myeloid cells 2; UCLH1, ubiquitin carboxyl-terminal esterase 1. *Denotes a *P*-value that survived FDR correction.

Bold indicates a biomarker that differentiated neurochemical AD from non-AD—significant after FDR correction in test and validation cohorts. Italics indicate a biomarker that differentiated neurochemical AD from non-AD—significant after FDR correction in test cohort only.

Denotes biomarkers also identified using OPLS-DA analysis where subjects were classified neurochemically.

**Table 2B:** Univariate analysis comparing biomarkers in AD and non-AD CSF (excluding healthy controls)

	P*-value (cohort 2)*	*Fold change*
**Malate dehydrogenase**	**<0.001**	**1.85**
V-Set and transmembrane domain containing protein 2A	0.001	1.71
LSAMP	0.003	1.65
Total APOE	<0.001	1.61
S100B	0.004	1.48
Chitinase-3-like protein 1 (YKL-40)	<0.001	1.47
Cystatin C	0.003	1.44
Osteopontin	0.03	1.43
LAMP1	0.008	1.42
CD166	0.02	1.40
Pro-orexin	<0.001	1.30
Beta-amyloid 40	<0.001	1.38
CNDP1	<0.001	1.38
Carboxypeptidase E	0.004	1.37
GM2	0.04	1.35
NCAM1	0.03	1.25

Abbreviations: AD, Alzheimer's disease; CNDP1, carnosine dipeptidase 1; CSF, cerebrospinal fluid; LSAMP, limbic system-associated membrane protein; NCAM1, neural cell adhesion molecule 1.

Bold indicates a biomarker that differentiated neurochemical AD from non-AD—significant after FDR correction.
