# Supplementary figures and images for: A targeted proteomic multiplex CSF assay identifies increased malate dehydrogenase and other neurodegenerative biomarkers in individuals with Alzheimer's disease pathology
Source: Transl Psychiatry. 2016 Nov 15;6(11):e952–. doi: 10.1038/tp.2016.194 (PMC5314115; doi:10.1038/tp.2016.194)

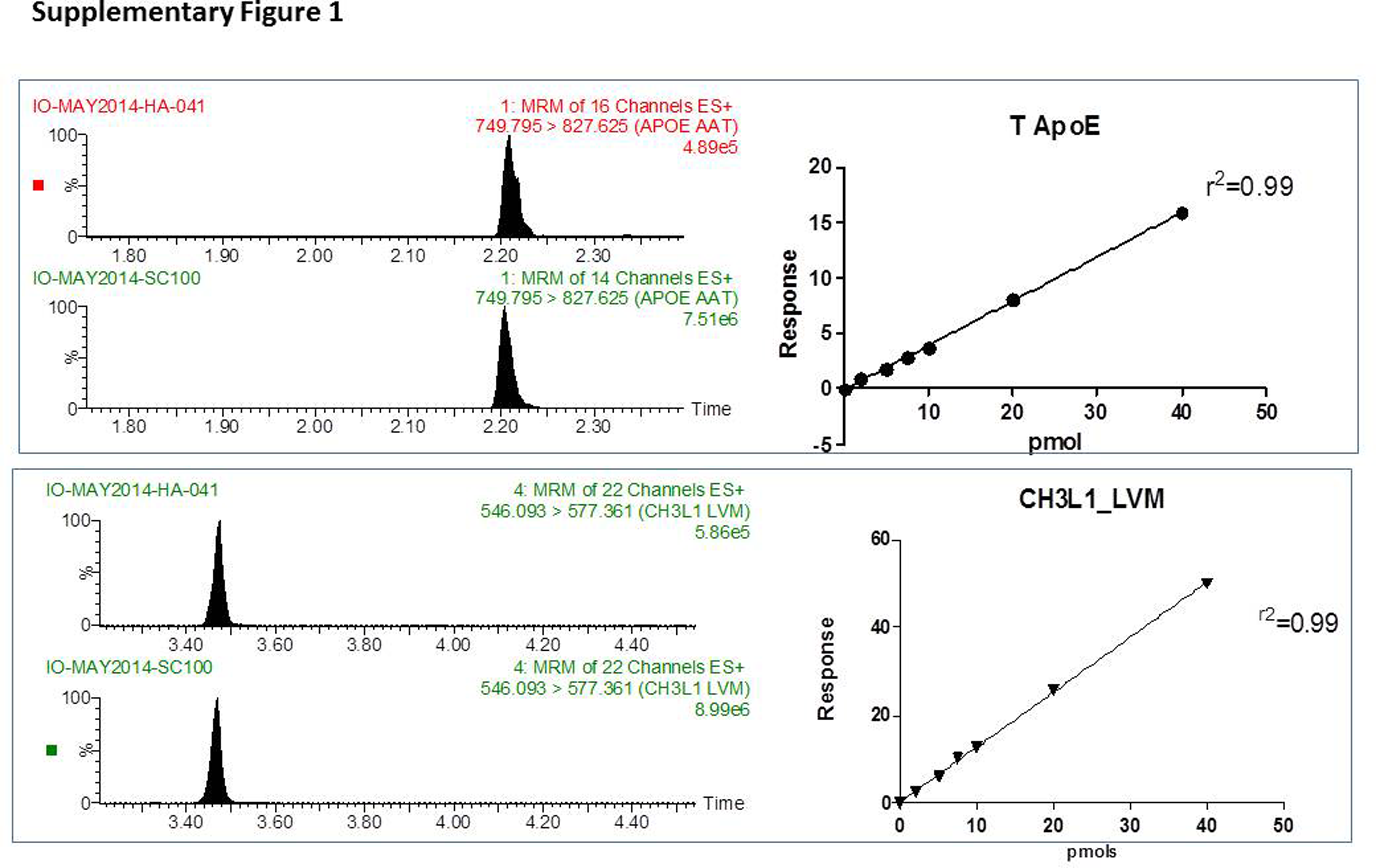

Supplement: Supplementary Figure 1 [file tp2016194x1.tif]

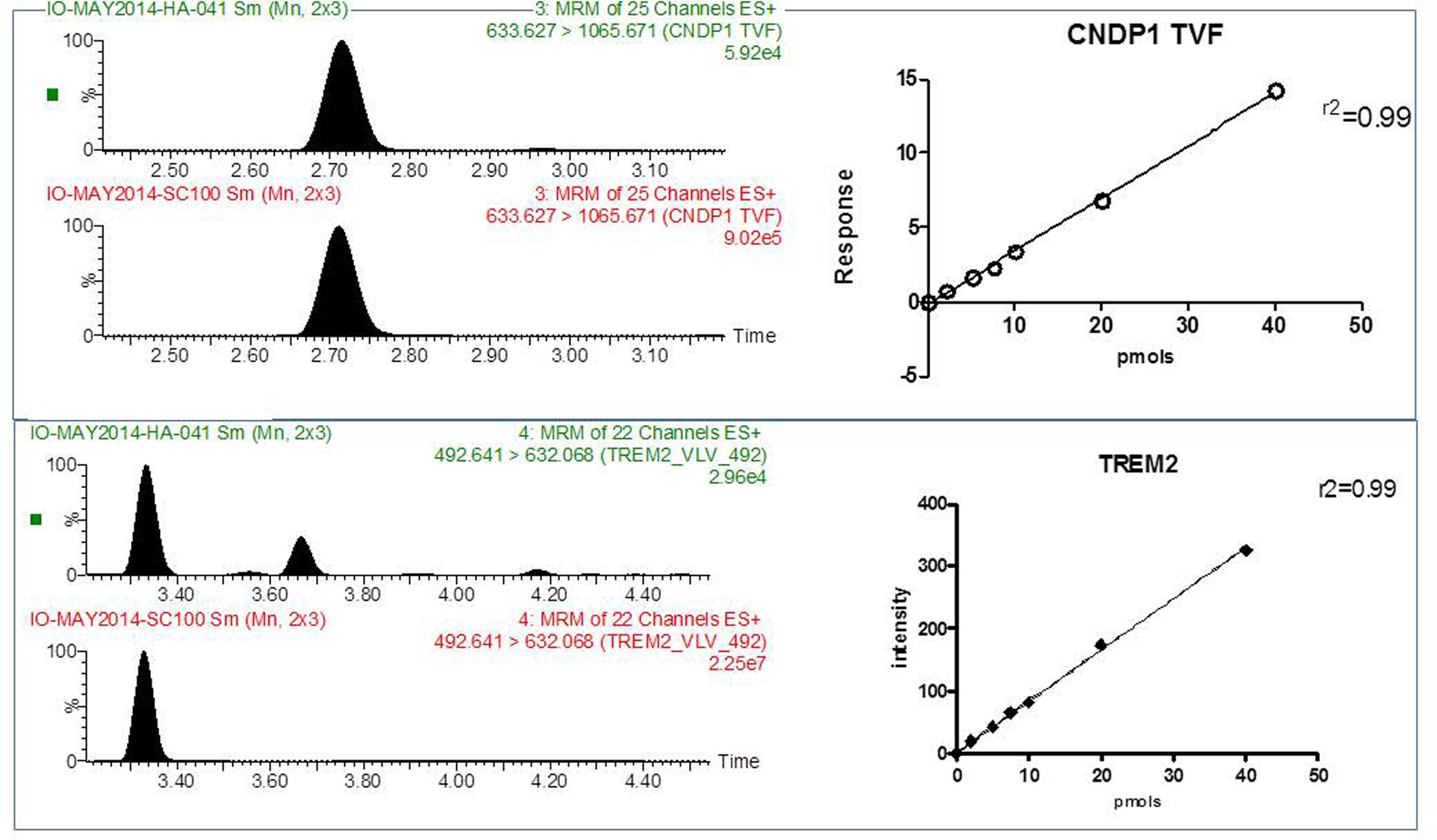

Supplement: Supplementary Figure 2 [file tp2016194x2.tif]

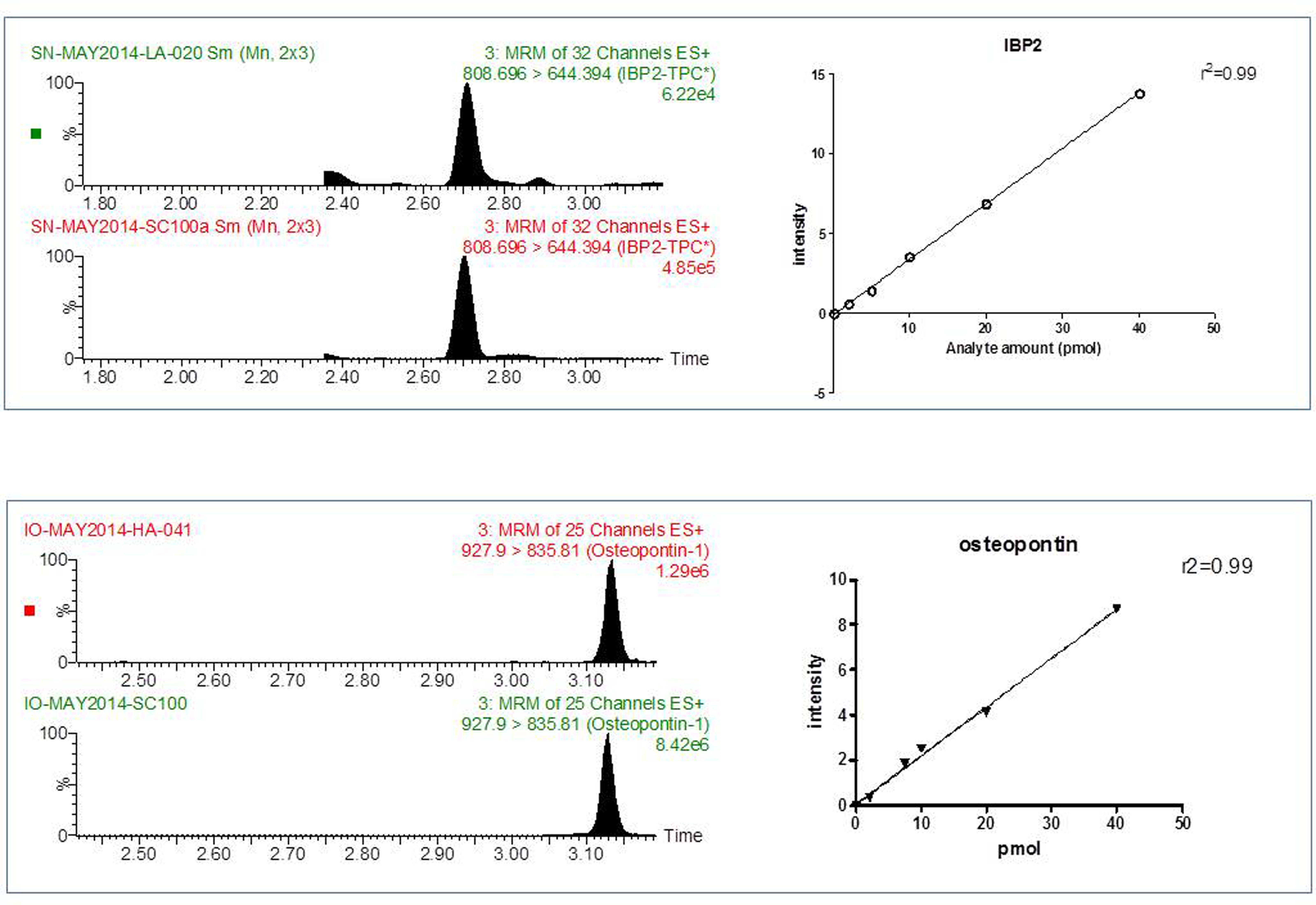

Supplement: Supplementary Figure 3 [file tp2016194x3.tif]

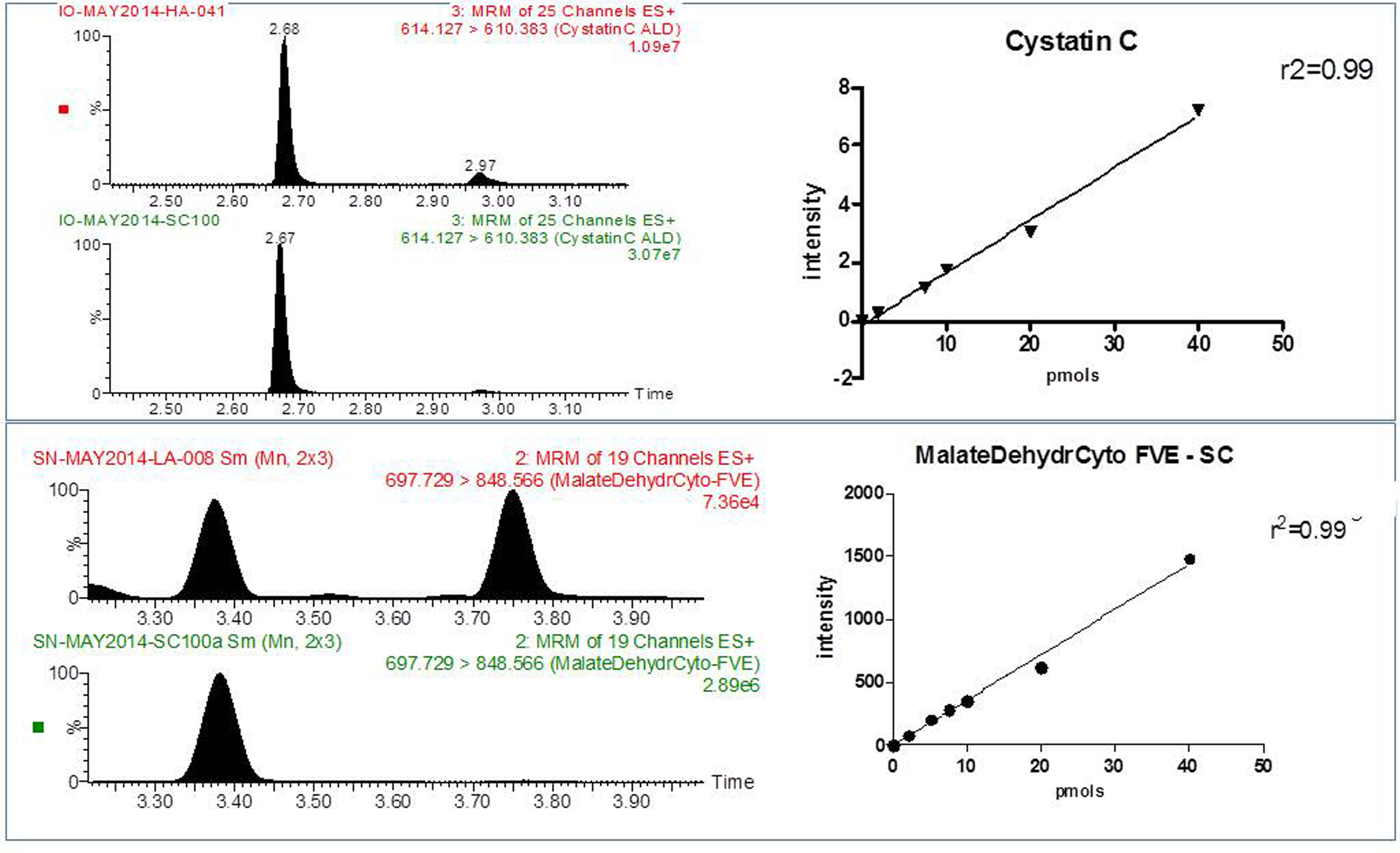

Supplement: Supplementary Figure 4 [file tp2016194x4.tif]

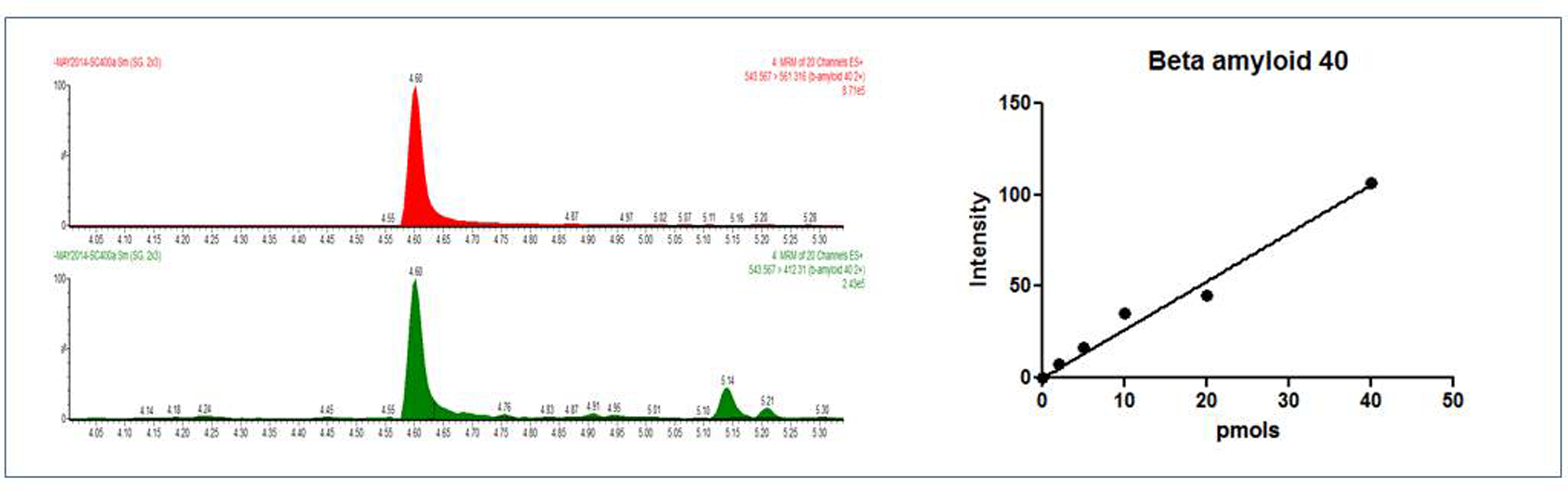

Supplement: Supplementary Figure 5 [file tp2016194x5.tif]
