# Supplementary material for: A targeted proteomic multiplex CSF assay identifies increased malate dehydrogenase and other neurodegenerative biomarkers in individuals with Alzheimer's disease pathology
Source: Transl Psychiatry. 2016 Nov 15;6(11):e952–. doi: 10.1038/tp.2016.194 (PMC5314115; doi:10.1038/tp.2016.194)
Supplement: Supplementary Table 1 [file tp2016194x6.pdf]

List of peptides and transitions included in Multiplex targeted proteomics assay

Name	Protein	Precursor m/z	Product m/z	Peptide sequence	Detectable in 100ul CSF (test cohort)?	Detectable in 100ul CSF (validation co
Apolipoprotein AII	APOA2 SPE	487.0511	659.4627	SPELQAEAK	yes	yes
	APOA2 SPE	487.0511	**788.5582**			
Apolipoprotein E (total)	APOE AAT	749.7949	642.4312	AATVGSLAGQPLQER	yes	yes
	APOE AAT	749.7949	**827.6254**			
Apolipoprotein E E2 isoform	ApoE E2 carbo	554.79	**345.14**	CLAVYQAGAR	yes	yes
	ApoE E2 carbo	554.79	835.5545			
Apolipoprotein E 112 E3	ApoE E3 112	611.76	**491.24**	LGADMEDVCGR	yes	not tested
	ApoE E3 112	611.76	606.27			
Apolipoprotein E 158 E3	APOE E3 158	475.0873	502.3396	LAVYQAGAR	yes	yes
	APOE E3 158	475.0873	**665.4433**			
Apolipoprotein E E4 isoform	APOE E4	503.5173	835.4926	LGADMEDVR	yes	not tested
	APOE E4	503.5173	892.499			
Apolipoprotein E E4 isoform Aqua peptide	APOE E4 AP	506.524	841.5126	AAQARLGADMED[V(13C5;	yes	yes
	APOE E4 AP	506.524	898.519			
Apolipoprotein H (beta2microglobulin)	ApoH non carbo	796.0762	**503.325**	ATFGCHDGYSLDGPEEIECTK	yes	yes
	ApoH non carbo	796.0762	531.799			
Carboxypeptidase E	CarboxypepE-ELL	817.2226	356.2624	ELLVIELSDNPGVHEPGEPEFI	yes	yes
	CarboxypepE-ELL	817.2226	**820.165**			
CD166	CD166-QIG*	838.2949	**397.0361**	QIGDALPVSCTISASR	yes	yes
	CD166-QIG*	838.2949	829.1371			
Chitinase 3 Like protein 1 (YKL40)	CH3L1 LVM	546.0934	439.7986		yes	yes
	CH3L1 LVM	546.0934	**577.3608**	LVMGIPTFGR		
Chitinase 3 Like protein 1 (YKL40)	CH3L1 TLL	761.7649	654.4573	TLLSVGGWNFGSQR	yes	yes
	CH3L1 TLL	761.7649	**1008.5999**			
Clusterin	CLUS_LFD	625.6611	**585.9436**	LFDSDPITVTVPVEVSR	yes	yes
	CLUS_LFD	625.6611	686.5015			
Beta-Ala-His dipeptidase	CNDP1 TVF	633.6272	533.4147	TVFGTEPDMIR	yes	yes
	CNDP1 TVF	633.6272	**1065.6713**			
Beta-Ala-His dipeptidase	CNDP1 WNY	506.0911	284.1387	WNYIEGTK	yes	yes
	CNDP1 WNY	506.0911	**497.3354**			
Cystatin C	CystatinC ALD	614.1272	300.2003		yes	yes
	CystatinC ALD	614.1272	**610.383**	ALDFAVGEYNK		
Ectonucleotide pyrophosphatase/phosphodiesterase fa	ENPP2 WWG*	772.4634	**929.4495**	WWGGQPLWITATK	yes	yes
	ENPP2 WWG*	772.4634	1171.4946			
Fibrinogen beta chain	FIBB_MGP_3p	846.65	333.18	MGPTELLIEMEDWK	yes	yes
Ganglioside GM2 activator	GM2_by_KM*	775.6234	769.6363		yes	yes
	GM2_optimum*	775.6234	**213.176**	SEFVVPDLELPSWLTTGNYR		
Insulin like Growth Factor 2	IGF2_SCD	906.9619	**315.2079**	SCDLALLETYCATPAK	yes	yes
	IGF2_SCD	906.9619	363.0458			
Lysosome-associated membrane glycoprotein 2	LAMP2_GIL_2P_02*	656.91	829.45		yes	yes
	LAMP2_GIL_2P_03*	656.91	**359.21**	GILTVDELLAIR		
Limbicsystem associated membrane protein	LSAMP Doubly	529.9043	**521.1044**	INSANGLEIK	yes	yes
	LSAMP Doubly	529.9043	831.6159			
Osteopontin	Osteopontin-1	927.9	511.36	AIPVAQDLNAPSDWDSR	yes	yes
	Osteopontin-1	927.9	**835.81**			
Pappalysin-1	PAPP A LDG 3+ ok	488.3037	365.4114	LDGSTHLNIFFAK	yes- not quantifiable due to peak interference	yes
	PAPP A LDG 3+ ok	488.3037	495.5653			
Prosaposin*	SAPA_EIV_2P_02	865.46	215.18	EIVDSYLPVILDIIK	yes	not tested
	SAPA_EIV_2P_03	865.46	**910.53**			
Serum Amyloid A4	SerumAmyloidA4-EAL	567.0596	363.2195	EALQGVGDMGR	yes	yes
	SerumAmyloidA4-EAL	567.0596	**535.3104**			
Apolipoprotein E Heavy peptide	TIMP1-GFQ	617.5534	404.1179	GFQALGDAADIR	yes	yes
	TIMP1-GFQ	617.5534	**717.3912**			
Metalloproteinase inhibitor 1	TREM2_VLV_492	492.6411	**632.0681**	VLVEVLADPLDHR	yes	yes
	TREM2_VLV_492	492.6411	688.6233			
Triggering receptor expressed on myeloid cells 2	TRFE_EFQ_3p	426.5	387.21			
	TTHY_AAD_2p	698.13	**606.36**	AADDTWEPFASGK	yes	not tested
	TTHY_YTI_3p	787.82	**1002.3**	YTIAALLSPYSYSTTAVVTNPK	yes	yes
Transthyretin	Ubqtn_TIT	894.7511	298.1879	TITLEVEPSDTIENVK	yes	No
Transthyretin	Ubqtn_TIT	894.7511	**1002.5528**			
Ubitqutin species derived peptide	VITAMIN D BINDING PROTEIN	789.54	**657.24**	VPTADLEDVLPLAEDITNILSK	yes	yes
	VITAMIN D BINDING PROTEIN	789.54	1053.93			
VITAMIN D BINDING PROTEIN	Vitrnctn FED*	712.1811	435.3108	FEDGVLDPDYPR	yes	yes
	Vitrnctn FED*	712.1811	**647.4367**			
Vitromectin	Vitrnctn SIA*	835.4319	310.2264	SIAQYWLGCPAPGHL	yes	yes
	Vitrnctn SIA*	835.4319	**423.2977**			
Vitromectin	VSetTM2-GPE*	1008.031	627.2576	GPEDLDPGAEGAGAQVELLPDR		
	VSetTM2-GPE*	1008.031	**1198.2634**		yes	yes
	Yeast Enolase AVD 789	790.1596	661.4872	AVDDFLISLDGTANK		
Vset transmembrane domain 2	Yeast Enolase AVD 789	790.1596	805.5706			
Yeast Enolase internal standard protein	Yeast Enolase AVD 789	790.1596	918.6694			
	Yeast Enolase GNP 708	709.0596	377.2864	GNPTVEVELTTEK		
	Yeast Enolase GNP 708	709.0596	451.4681			
	Yeast Enolase GNP 708	709.0596	948.6764			
	Yeast Enolase SIV 614	614.5796	306.2782			
	Yeast Enolase SIV 614	614.5796	514.8752	SIVPSGASTGVHEALEMR		
	Yeast Enolase SIV 614	614.5796	547.8778			
	Yeast Enolase SIV 614	614.5796	821.3544			

*Denotes biomarkers identified using 2D-LC-MSe profiling of neurodegenerative CSF
Red colour denotes peptides that could not reliably be detected in CSF.

Name	Protein	Precursc	Product	Peptide sequence	Detectable in 100ul CSF?	
b-amyloid 40	b-amyloid 40 2+	543.5673	412.31			
	b-amyloid 40 2+	543.5673	**561.3157**	GAIIGLMVGGVV	yes	yes
Triggering receptor expres	TREM2PEP1	562.01	**937.18**	VVSTHNLWLLSFLR	yes	yes
	TREM2PEP1	562.01	1050.27			
Neural Cell Adhesion Mole	NCAM1 FIV	662.6772	532.9085	FIVLSNNYLQI	yes	not tested
	NCAM1 FIV	662.6772	**597.0048**			
Trefoil 3	trefoil3_IPG	726.8572	715.201	IPGVPWCFKPLQEAECTF	no	not tested
	trefoil3_IPG	726.8572	721.1159			
glucosylceramidase 1	GBA1-2_Doubly	731.16	**1100.6**	NFVDSPIIVDITK	yes	no
	GBA1-2_Doubly	731.16	1199.67			
Insulin like Growth Factor	IGF1-GPE	770.1011	**347.1612**	GPETLCGAELVDALQFVCGDR	yes	yes
	IGF1-GPE	770.1011	606.2991			
Pappalysin-1	PappA VSF	802.0043	**786.5862**	VSFSSPLVAISGVALR	yes	yes
	PappA VSF	802.0043	1095.86			
Lysosome-associated mem	LAMP1_FFLQGIQLNTILPDAR	923.9249	458.272	FFLQGIQLNTILPDAR	yes	yes
	LAMP1_FFLQGIQLNTILPDAR	923.9249	**571.3269**			
Myelin basic protein	MBP_TQD	487.8949	285.1158	TQDENPVVHFFK	no	not tested
	MBP_TQD	487.8949	616.4953			
Ubiquitin carboxyl-termina	UCHL1-LGF	532.7281	**747.3007**	LGFEDGSVLK	yes	no
	UCHL1-LGF	532.7281	894.3056			
DJ1	DJ1_triply	554.2234	674.1549	GLIAAICAGPTALLAHEIGFGSK	yes	no
	DJ1_triply	554.2234	**723.7188**			
Serum Amyloid P	SerumAmyloidP	578.9681	508.3865	VGEYSLYIGR	yes	yes
	SerumAmyloidP	578.9681	**708.5315**			
Ras-related protein Rab-3(	Rab30_QNT 3+	585.2757	442.2915	QNTLVNNVSSPLPGEGK	yes	yes
	Rab30_QNT 3+	585.2757	**487.2676**			
	Yeast Enolase SIV 614	614.5796	306.2782			
	Yeast Enolase SIV 614	614.5796	514.8752			
	Yeast Enolase SIV 614	614.5796	547.8778			
	Yeast Enolase SIV 614	614.5796	821.3544			
Ubiquitin carboxyl-termina	UCHL1-MPF	615.1096	**734.5051**	MPFPVNHGASSEDTLLK	yes	no
	UCHL1-MPF	615.1096	856.5666			
alpha synuclein	A syn quadru	643.7273	**339.3462**	EQVTNVGGAVVTGVTAVAQK	yes	no
	A syn quadru	643.7273	346.3015			
Tau protein (MAPT)	T-Tau LQT	655.7134	**472.3165**	LQTAPVPMPDLK	yes	no
	T-Tau LQT	655.7134	896.6035			
Tau protein (MAPT) heavy	T-Tau LQT AP	659.2219	**479.3165**	SRLQTAPVPMPD[L(13C6; 15N)]K		
	T-Tau LQT AP	659.2219	903.6035			
	GBA2 ACG	691.522	743.1383			
	GBA2 ACG	691.522	836.2809			
Aldolase B	AldoB-IAD	715.1687	709.1391	IADQCPSSLAIQENANALAR	no	not tested
	AldoB-IAD	715.1687	858.7665			
Synapsin 1	Synapsin 1	727.7049	**443.3207**	EMLSSTTYPVVVK	Not very w	yes
	Synapsin 1	727.7049	541.5081			
Neural Cell Adhesion Mole	NCAM1 YIF	759.2849	1093.695	YIFSDDSSQLTIK	yes	yes
	NCAM1 YIF	759.2849	**1240.867**			
Insulin-like growth factor-l	IBP2-TPC*	808.6957	644.3944	TPCQQELDQVLER	yes	yes
	IBP2-TPC	808.6957	**758.3367**			
b-amyloid 38	b-amyloid 38 2+	444.4611	**363.1562**	GAIIGLMVGG	yes	yes
	b-amyloid 38 2+	444.4611	525.3007			
Endothelial protein C rece	EPCR-TLA	516.0596	**433.2602**		yes	no
	EPCR-TLA	516.0596	816.6147	TLAFPLTIR		
protein S100B	S100B	569.9873	**703.7626**	AMVALIDVFHQYSGR	yes	yes
	S100B	569.9873	753.2795			
Neurofilament light protei	NFL-VLE	578.2011	**387.2939**	VLEAELLVLR	not very w	not tested
	NFL-VLE	578.2011	942.7229			
Insulin B	InsB digest 651 4+	651.4043	521.3715	FVNQHLCGSHLVEALYLVCGER	no	not tested
	InsB digest 651 4+	651.4043	797.3206			
	GBA2 NVI	663.5311	514.3973			
	GBA2 NVI	663.5311	888.35			
Malate dehydrogenase	MalateDehydrCyto-FVE	697.7287	546.3637	FVEGLPINDFSR	yes	yes
	MalateDehydrCyto-FVE	697.7287	**848.5662**			
	Yeast Enolase AVD 789	790.1596	661.4872			
	Yeast Enolase AVD 789	790.1596	805.5706	AVDDFLISLDGTANK		
	Yeast Enolase AVD 789	790.1596	918.6694			
Lysosome-associated mem	LAMP1_NMTFDLPSDATVVLNR_897	897.3534	364.1983	NMTFDLPSDATVVLNR	yes	yes
	LAMP1_NMTFDLPSDATVVLNR_897	897.3534	1071.726			
Trefoil 3	Trefoil3_vdc	424.9557	529.736	VDCGYPHVTPKECNNR	no	not tested
	Trefoil3_vdc	424.9557	587.2962			
Pro-orexin	14 OREXIN PROPEP TC	451.4787	**512.5109**	AGAEPAPRPCLGR	yes	yes
	14 OREXIN PROPEP TC	451.4787	641.1357			
progranulin	PRGN_VHC	492.2181	237.1425	VHCCPHGAFCDLVHTR	no	not tested
	PRGN_VHC	492.2181	413.234			
chitotriosidase	CHITO_ADG_2P_02	501.65	**311.2**	ADGLYPNPRER	not very w	yes
	CHITO_ADG_2P_01	501.65	646.2			
Fibrinogen A	FIBA_HPD_2p	532.19	621.25	HPDEAAFFDTASTGK	yes	yes
Glial fibrillary acidic protei	GFAP	589.5873	**616.4967**	LADVYQAELR	yes	no
	GFAP	589.5873	779.5966			
Gltathione S transferase o	GSTO1-GSA	661.1811	**553.3864**	GSAPPGPVPEGSIR	yes	yes
	GSTO1-GSA	661.1811	658.3671			
	Yeast Enolase GNP 708	709.0596	377.2864	R.GNPTVEVELTTEK.G [15, 27]		
	Yeast Enolase GNP 708	709.0596	451.4681			
	Yeast Enolase GNP 708	709.0596	948.6764			
Trefoil 2	Tref_Fac2_QES	791.7243	377.1338	QESDQCVMEVSDR	no	not tested
	Tref_Fac2_QES	791.7243	782.7479			
Trefoil 2	TREF_FAC2_NCG	848.8381	332.1084	NCGYPGISPEECASR	no	not tested
	TREF_FAC2_NCG	848.8381	1202.727			
